# Plumbagin, a Natural Compound with Several Biological Effects and Anti-Inflammatory Properties

**DOI:** 10.3390/life13061303

**Published:** 2023-05-31

**Authors:** Giovannamaria Petrocelli, Pasquale Marrazzo, Laura Bonsi, Federica Facchin, Francesco Alviano, Silvia Canaider

**Affiliations:** 1 Department of Medical and Surgical Sciences, University of Bologna, 40126 Bologna, BO, Italy; giovannam.petrocell2@unibo.it (G.P.); laura.bonsi@unibo.it (L.B.); federica.facchin2@unibo.it (F.F.); silvia.canaider@unibo.it (S.C.); 2 Department of Biomedical and Neuromotor Science, University of Bologna, 40126 Bologna, BO, Italy; francesco.alviano@unibo.it

**Keywords:** plumbagin, plumbaginaceae, natural compound, inflammation, anti-inflammatory, antioxidant, antibacterial, antiparasitic, anti-senescence, stem cells

## Abstract

Phytochemicals from various medicinal plants are well known for their antioxidant properties and anti-cancer effects. Many of these bioactive compounds or natural products have demonstrated effects against inflammation, while some showed a role that is only approximately described as anti-inflammatory. In particular, naphthoquinones are naturally-occurring compounds with different pharmacological activities and allow easy scaffold modification for drug design approaches. Among this class of compounds, Plumbagin, a plant-derived product, has shown interesting counteracting effects in many inflammation models. However, scientific knowledge about the beneficial effect of Plumbagin should be comprehensively reported before candidating this natural molecule into a future drug against specific human diseases. In this review, the most relevant mechanisms in which Plumbagin plays a role in the process of inflammation were summarized. Other relevant bioactive effects were reviewed to provide a complete and compact scenario of Plumbagin’s potential therapeutic significance.

## 1. Introduction

Over the years, a plethora of biocompounds derived from plants have been used for human healthcare owing to their antimicrobial, antioxidant, anticancer, and anti-inflammatory effects. Many biocompounds are usually plant secondary metabolites, whose biological features make them interesting for human supplementation and the development of new drugs. Among the plant secondary metabolites, naphthoquinones are the largest group [1]. Naphthoquinones are aromatic cyclic compounds that exert important pharmacological activities, such as anticancer [2] and antifertility [3], as well as broad antimicrobial [4], antibacterial [5], and anti-inflammatory effects [6]. The mechanism of action of naphthoquinones is mainly dependent on the redox state of the cells. In fact, these biocompounds inhibit the electron transport during oxidative phosphorylation and generate reactive oxygen radicals in aerobic conditions. At the molecular level, naphthoquinones act also as alkylating and intercalating agents in the DNA double helix [7].

Plumbagin (PB) is a naphthoquinone obtained from the roots of different medicinal plant families, such as *Plumbaginaceae*, *Droseraceae*, and *Ebenaceae*. *Plumbago zeylanica* L. has been considered the most known medicinal plant that contains PB [8,9]. Chemically, PB is structurally similar to vitamin K, while its half-life is rather short [10]. As for most plant-derived molecules, PB shows low solubility in water and, critically, limited oral bioavailability [11] as well as moderate toxicity [12]. Despite that, it still appears to be an intriguing candidate for the development of new therapeutic agents [13,14]. Several studies have shown the biological activities of this molecule, such as those antioxidant, antibacterial, antifungal, analgesic, anti-inflammatory, and anticancer [7,15,16]. In literature, the anticancer effect of PB has been deeply investigated [13,17], while there are no recent reviews focused on the anti-inflammatory activity of this compound.

Inflammation is a process underlying all chronic diseases, such as cancer, autoimmune disease, cardiovascular, and neurodegenerative ones. Over the years, several in vitro and in vivo studies have been carried out on inflammatory models to understand the mechanism underpinning the anti-inflammatory activity of PB. The studies showed how PB can be implicated in inflammation signaling by blocking Nuclear factor-kB (Nf-kB) and Mitogen-activated protein kinase (MAPK) pathways (throughout p38 and c-Jun N-terminal kinase (JNK) cassettes) [18,19,20]. This review aimed to summarize the current knowledge on the anti-inflammatory activity of PB, as well as on the cytoprotective and anti-senescent actions, with the goal of providing a comprehensive overview of all potential therapeutic applications of this biocompound, including in the regenerative medicine field.

## 2. The Cytoprotective Activity of PB

The cytoprotective activity of PB is strictly related to its antioxidant activity. In the literature, there are many studies that show PB cytoprotective activity, both in vitro and in vivo models. PB exerts its protective role against H_2_O_2_-induced damage in several cell models [21,22]. Pre-treatment with PB for 24 h in neuronal cells [22] or chondrocytes [19] exposed to H_2_O_2_ promoted cell viability to 100%. The increase in cell viability after PB pre-treatment was associated with an enhancement in antioxidant enzyme activities (superoxide dismutase—SOD, catalase—CAT, glutathione S-transferase—GST, glutathione peroxidase—GPx) and the expression of nuclear factor erythroid 2-related factor 2 (Nrf-2) [21,22,23]. Importantly, Nrf-2 plays a pivotal role in cellular resistance to oxidative agents. Those results have been replicated also in osteoblasts [24]. Indeed, PB pre-treatment increased cell viability (around 90%) and downregulated the expression of caspases 3, 8, and 9 in association with an increase in Nrf-2 expression [24]. The cytoprotective activity of PB occurred not only when it was administrated as pre-treatment, but also after the administration of H_2_O_2_, as demonstrated by Chu and co-workers, on rat nucleus pulposus cells [25]. Among the cytoprotective activities, PB exerts the antiapoptotic one. This is also related to a downregulation of the pro-apoptotic protein Bcl-2-Associated X-protein (Bax) and an upregulation of the anti-apoptotic protein B-cell lymphoma 2 (Bcl-2) [26]. The pathways implied in the anti-apoptotic effect of PB have also been validated in an ischemia mouse model [27]. It is well-known that apoptosis is strictly related to mitochondrial membrane potential, since a marked reduction of this potential leads to cell apoptosis. It has been shown that dexamethasone induced apoptosis in osteoblasts by reducing mitochondrial membrane potential and that this effect was restored by treatment with PB [24]. Similar results have been obtained on oxygen-glucose deprivation/reoxygenation (OGDR)-induced neuroinjury in human SH-SY5Y 2D [28] and a 3D culture model [29]. In vivo experiments, in addition to the neuroprotective [27] role of PB, showed hepatoprotective potential [30]. By using a murine model of acute liver injury, Wang and colleagues found enhanced liver regeneration after PB administration. This was related to an increase in hepatocyte proliferation and reduced apoptosis levels [30]. Autophagy as a protective mechanism induced by PB was reported by Pan and colleagues in rat hepatic tissue of bile duct ligation (BDL)-induced cholestatic liver injury [31].

Recently, researchers have shown interest in the potential cardioprotective role of PB. Cardiovascular disease is the world’s leading cause of death [32]. Among the several causes, this disease is triggered by chemotherapeutic agents, such as doxorubicin. Doxorubicin-mediated cardiovascular toxicity is a restricting factor that limits the wide usage of this chemotherapy drug [33]. Recently, Li and co-workers showed an interesting protective role of PB against doxorubicin-stimulated cardiotoxicity in rats [34]. PB treatment reduced the circulant levels of cardiac damage markers (lactate—LDH, aspartate aminotransferase—AST, and creatine kinase—CK) in serum of PB-supplemented animals. Moreover, in rats, PB enhanced the expression of phosphatase and tensin homolog (pTEN) protein and inhibited the phosphoinositide-3-kinase/protein kinase B or Akt (PI3K/Akt) signaling pathway that was able to activate the apoptosis cascade [34]. The inhibition of apoptosis and ROS levels following PB treatment in cardiomyocytes was also investigated by Zhang and co-workers [35] by using H9c2 cardiomyocytes. In this study, the cytoprotective effect of PB was investigated in a model of oxidative stress induced by tertiary butyl hydrogen peroxide (THBP). Pre-treatment with PB increased cell viability, and decreased both ROS levels and apoptotic cell rate, according to the data of previous works [21,22,24]. The novelty of this work was the identification of a new mechanism of action whereby PB reduces apoptosis. In fact, the authors demonstrated an increase in the autophagic process in their in vitro model. In particular, the autophagic activity was stimulated by the inhibition of the p38 MAPK pathway, increasing microtubule-associated protein light chain 3 (LC3)II/LC3I ratio [35].

So far, the data obtained are encouraging. However, more in vivo studies must be carried out in order to ensure PB as a cytoprotective molecule in therapeutic applications.

## 3. The Anti-Inflammatory Activity of PB

As mentioned above, over the years the anti-inflammatory role of PB has gained interest due to a putative implication in the therapeutic field. Inflammation can be characterized by an imbalance of both reactive oxygen/nitric species (ROS/RNS) and pro-inflammatory cytokines. The equilibrium between pro-oxidant ROS or RNS and antioxidant agents (such as CAT, GPx, and SOD), cellular metabolism, and respiration determines the redox homeostasis of the cells. Redox status is also represented by the endogenous antioxidant glutathione GSH/GSSG ratio, which is central also for the immune system function [36]. Here, glutathione level plays a pivotal role in the activation, proliferation, and survival of cells. Checker and collaborators suggested that PB may induce a change in the redox status of murine lymphocytes [37]. In this cellular model, PB determined an increase in ROS levels and free thiols and altered the glutathionylation state of the proteins by binding the GSH. Moreover, after the PB treatment, the Nf-kB factor resulted in a glutathionylated form that prevented its nuclear translocation and the following activation of the survival cascade in the lymphocytes [37]. The same mechanism of action was described by Wang and colleagues in 2014 by using a RAW264.7 cellular model [19] and by Checker and co-worker in a mouse model of endotoxic shock [20]. In this model, the administration of endotoxins usually caused lethality in 80–90% of cases, while treatment with PB 2 h prior to lipopolysaccharides (LPS) administration increased the survival rate to approximately 90%. PB treatment reduced the circulating levels of pro-inflammatory cytokines such as tumor necrosis factor (TNF) α, interleukin (IL) 6, and IL1 β, as well as nitric oxide (NO). Moreover, the histopathological analysis showed an improvement in granulocyte and lymphocyte infiltration in the liver and lungs, respectively [20]. PB protection against sepsis and endotoxic shock was also validated by Zhang and colleagues [38]. The researchers investigated the protective effect during inflammation of PB on bone marrow-derived macrophages: PB inhibited the activation of pyruvate kinase M2 (PKM2) enzyme, necessary for the metabolic switch from oxidative phosphorylation to glycolysis, which usually occurs in the activated macrophages. In the sepsis mouse model, after PB treatment, a reduction of IL1 β levels, but also high mobility group box 1 (HMGB1) (late intermediate of inflammation) [38], was observed. All things considered, the anti-inflammatory action of PB seems to be related to its pro-oxidant role. By reducing free GSH levels, PB increased the production of ROS and Nf-kB oxidation, preventing its binding to DNA [20]. The nuclear translocation of Nf-kB is also related to the absence of NF-kappa-B inhibitor alpha (IkBa) protein. During the inflammatory response, IkBa is degraded. PB stabilized IkBa, preventing Nf-kB nuclear translocation [18,20]. Arruri and collaborators showed that PB treatment on a rat model of neuropathic pain led to a decrease of NO levels, as previously described [18,20], and of pro-inflammatory cytokines by inhibiting the Nf-kB pathway and activating Nrf-2 [39].

PB counteracts inflammation consequences not only by acting as a pro-oxidant effector. In fact, several works showed the antioxidant effect of this natural molecule in the context of inflammation models. Research on the anti-inflammatory effect of PB was performed on microglia [40], and it is known to stimulate an excessive amount of pro-inflammatory cytokines if activated for a long time, leading to the development of neurodegenerative disorders [41]. LPS-activated microglia showed an increased expression of inducible nitric oxide synthase (iNOS) [42] and of cytokines like IL1 α, granulocyte-colony stimulating factors (G-CSF), IL12, monocyte chemoattractant protein (MCP) 1, and MCP5 [40]: all these effects were reverted by PB treatment [40]. In human SH-SY5Y exposed to OGDR, PB exerted a protective role by inactivating the NLR family pyrin domain containing 3 (NLRP3)-induced inflammasome [28]. The antioxidant role of PB was linked to the upregulation of GSH and GPx enzyme levels [26] and to the inactivation of the Nf-kB/TNF α pathway [26,27]. Recently, Mahmoud and colleagues suggested an additional mechanism of PB as an anti-inflammatory modulator. According to their studies, the anti-inflammatory action of PB was associated with the inactivation of the interleukin-1 receptor-associated kinase (IRAK1), which, in physiological conditions, is implied in the production of IL8, IL6, IL1 β, and TNF α, [43] which are chemokines with a fundament role in inflammation onset.

### 3.1. PB in Osteoarthritis, Rheumatoid Arthritis, and Osteoporosis

Osteoarthritis is a chronic inflammatory disorder that is mainly found in aged people but is also linked to some disorders, such as obesity or a sedentary lifestyle. Prolonged oxidative stress in the chondrocytes leads to cartilage degeneration [44]. The identification of natural molecules that could be able to modulate oxidative stress and prevent the onset of this pathology is an intriguing field of research. Guo and co-workers investigated in vitro the antioxidant effect of PB on chondrocytes [21]. After H_2_O_2_ treatment, PB decreased ROS levels and lipid peroxidation and increased the enzymatic activity of GSH, SOD, GST, CAT, and GPx. This was related to the downregulation of Nf-kB, pro-inflammatory cytokines (TNF α, IL18, IL6), cyclooxygenase 2 (COX2), and iNOS, and the upregulation of Nrf2, an inducer of expression in many antioxidant enzymes [21,45]. However, there is still little in vivo evidence of the beneficial effect of PB for osteoarthritis. Therefore, further investigations are necessary to better understand the possible implications of PB in the treatment of this pathology.

Many recent studies highlighted the great potential of PB for the treatment of rheumatoid arthritis (RA), an autoimmune disease associated with chronic inflammation. Among the cytokines implied in the development of this disease, RANK-ligand (RANKL) and IL34 are the most relevant [46,47], playing a pivotal role in remodeling bone. Alteration of their expression can lead to bone loss and chronic inflammation. It was seen that RANKL exerted an osteoclastogenic action and was inhibited by osteoprotegerin (OPG) [48]. Patients with RA showed an increased expression of RANKL and a decreased expression of OPG, thus leading to bone loss [49,50]. RANKL secretion was also related to the expression of IL34. IL17 is a crucial factor regulating the expression of IL34. PB has already been shown to be an IL17 inhibitor [51]. These findings were confirmed by Cui and co-workers. Indeed, the suppression effect of PB on IL17 in synoviocytes derived from RA patients decreased the effects mediated by IL34 [52]. As a result, after PB treatment, synoviocytes showed a decreased expression of RANKL and an increased expression of OPG [52] These findings unraveled the potential use of PB for the treatment of this pathology; further investigations need to be conducted to better understand the underlying molecular mechanism of action of PB.

RA represents also a risk factor for osteoporosis. Osteoporosis usually occurs in women after menopause. However, it can be induced by prolonged treatment with glucocorticoids, since 30–50% of chronically treated patients develop this pathology [53]. In vitro studies on osteoblasts treated with dexamethasone provided downregulation models of osteogenic markers, such as osteocalcin (OCN), osteopontin (OPN), and Runt-related transcription factor 2 (Runx2) [24]. Pre-treatment with PB upregulated the expression of the osteogenic markers, suggesting a potential application of this molecule for the study of osteoporosis [24]. Bone remodeling is related to the Nf-kB pathway and the inflammatory level of the cell. Nf-kB promoted the osteoclastogenic process by inducing RANKL expression [54,55,56]. Shen and colleagues investigated the effect of PB on osteoclastogenic differentiation [57], demonstrating the involvement of Nf-kB during the process. PB inhibited the action of neutrophil inhibitory factor (NIF), a key factor in the processing of Nf-kB p100 subunit into p52 form, thus resulting in the indirect inhibition of osteoclastic differentiation [57]. A low dosage of PB (0.1–0.3 μM) on bone marrow mesenchymal stem cells increased Runx2 and alkaline phosphatase (ALP) expression but did not affect osterix (OSX) expression, which is an early osteogenic promoter of differentiation. Differently, PB 1 μM inhibited osteogenic differentiation. Hence, PB may promote late osteogenic differentiation and osteoblast maturation in a way that can be dosage-dependent. The authors also obtained interesting results in vivo by using an ovariectomized mouse model with bone impairment: PB-treated mice showed an increase in the thickness of trabeculae and in the number of osteoblasts [57]. Further studies need to be conducted in order to understand the optimal dosage of PB that could promote osteogenic differentiation in vivo and the possibility to use this molecule for therapeutical and regenerative medicine.

Sultanli and co-workers highlighted limitations regarding the usage of PB. According to the investigated cellular model, PB can alternatively promote or inhibit osteoclastic differentiation [58]. PB has been tested on macrophages derived from two different mouse models, C57BL/6 and BALB. In C57Bl/6-derived macrophages, PB promoted the osteoclastic differentiation, by upregulating acid phosphatase 5 (Acp5), Cathepsin-k (Ctsk), and osteoclast-associated Ig-like receptor (Oscar) genes. In BALB-derived macrophages, PB reduced osteoclastic differentiation by inhibiting the nuclear factor of the activated T cell 1 (NFatC1) pathway [58]. In addition, the C57Bl/6-derived macrophages tolerated elevated doses of PB (up to 2 µM), while in the BALB-derived macrophages, the same dosage was toxic for the cells. The authors hypothesized this opposite action was due to the different genetic backgrounds of the mice. Therefore, in the pre-clinical evaluation of PB as a therapeutic treatment for bone diseases, researchers should consider the influence of genetic background and the different responses to inflammation of the employed animal models.

### 3.2. The Antifibrotic Activity of PB

Fibrosis refers to the formation of new fibrotic connective tissue following damage or trauma in an organ. The deposition of fibrotic tissue leads to a loss of function in the damaged organ. The increase in collagen and in extracellular matrix levels is often related to the presence of an inflammatory process, that, if persistent, leads to impaired tissue function. Due to this correlation, researchers have focused their attention on the possible implication of PB in the treatment of fibrotic tissues. Wei and co-workers investigated the effects of PB both in vitro and in vivo [59]. For the in vivo studies, they used a model of carbon tetrachloride (CCl4)-induced hepatic fibrosis. CCL4 increased the circulating levels of alanine transaminase (ALT), AST, IL6, and TNF α and this upregulation was suppressed by PB administration. In vitro studies, PB reduced collagen deposition and alpha-smooth muscle actin (α-SMA) expression in hepatic stellate cells [59]. The same in vivo model was used by Chen and colleagues to investigate the molecular pathways involved in PB action [60]. Here, PB inactivated the epidermal growth factor receptor (EGFR) as well as the signal transducer and activator of transcription 3 (STAT3) signaling. With the same mouse model, a few years ago, Chen and co-workers showed how the antifibrotic activity of PB was connected to its antioxidant properties: PB increased the enzymatic activity of SOD, GSH, and GPx, leading to a reduction of collagen III deposition and α-SMA protein expression [61]. Similar findings were obtained in thioacetamide-induced hepatic fibrosis, where PB inhibited the Nf-kB and Akt pathways in the hepatic stellate cells, blocking the collagen deposition [30]. In addition to the α-SMA protein expression, PB downregulated the transforming growth factor (TGF)1-β protein expression in myofibroblasts and hepatic stellate cells [31].

The antifibrotic activity of PB was studied with success in models of lung fibrosis. The Bleomycin-induced lung fibrosis caused tissue structural damage, inflammation, and collagen deposition by fibroblasts [62]. Moreover, PB seems to inhibit the TGF β pathway, as demonstrated in other works [62,63,64]. A more in-depth analysis clarified the correlation between PB and TGF-β. PB regulated this factor at the epigenetic level by blocking the histone acetyltransferase p300 [62]. As a consequence, all the genes related to collagen production (such as col1a1, col3a1, α-SMA) were not expressed [62]. In addition to the TGF β pathway, PB also modulated mammalian targets of rapamycin (mTOR) and Akt [64].

### 3.3. Antibacterial and Antiparasitic Activity of PB

Over the years, the bacterial evolution and the genetic mutations together with the excessive and abusive usage of antibiotics led to an increase in antibiotic resistance acquired by several pathogenic microorganisms [65,66]. Consequently, there is a pressing need of investigating new antimicrobial agents. In the literature, several reviews regarding the antimicrobial, antimycotic, and antiparasitic effects of PB were written [1,67,68]. Here, we summarized the most recent works that described the molecular mechanisms underlying PB activities.

Several studies demonstrated that PB, in association with antibiotics or antimycotics, allowed reducing drug dosage and increasing their efficacy [69,70,71]. For example, the synergistic effect of PB with commercial drugs was investigated in *K. pneumoniae* [70]. By combining PB with gentamicin antibiotic, the dosage of gentamicin decreased from 16 µg/mL to 4 µg/mL. PB augmented the efficacy of gentamicin by promoting the intracellular uptake of the drug into the pathogen [70]. Moreover, the efficacy of PB was also studied on methicillin-resistant *S. aureus* MRSA [72]. Dissanayake and colleagues described an antimicrobial mechanism of action of PB in S. *aureus*: PB inhibited DNA gyrase activity, a topoisomerase relevant for the pathogen duplication, by binging the enzyme active site [73]. Interesting results were obtained in the treatment of colistin-resistant *P. aeruginosa* [71]. The usage of colistin antibiotics has been limited since this drug is nephrotoxic. Researchers combined PB with colistin and observed a synergistic effect stopping biofilm formation. In particular, PB increased pathogen susceptibility to colistin by altering the membrane permeability [71]. A similar alteration of membrane permeability following PB treatment was previously reported by Reddy and colleagues in *B. subtilis* [74].

In 2020, Sarkar and colleagues proposed an additional mechanism of action for PB. When *M. tuberculosis* was treated with PB, PB bound and inhibited the activity of thymidylate synthase X (ThyX), an enzyme implicated in bacterial duplication and is necessary for dTMP synthesis, starting from dUMP [75].

The antiparasitic activity of PB was also tested on *Leishmania* [76] and *C. elegans* [77], where PB perturbated the mitochondrial membrane potential and reduced the number of mitochondria, respectively.

A limit of PB usage consists of its poor solubility in water [11]. Rashidzadeh and co-workers synthesized nanoparticles to optimize the PB delivery and its bioavailability for the treatment of malaria. The nanoparticles carrying PB were tested in vivo on mice infected with *P. berghei*. The obtained data showed a better antiplasmodial activity of PB-loaded micelles in comparison with free PB activity [78]. In addition, this study showed the great potential of the nanoparticles as carriers for PB.

## 4. The Role of PB in Stem Cells and in Cell Senescence

While the anti-inflammatory effect of PB has been extensively studied, the effect of this natural compound on stem cells or other progenitor cell types is still unknown. Several studies have investigated the antitumoral effect of PB [13,79], specifically focusing on PB and cancer stem cell (CSC) biology. CSCs are responsible for the abnormal growth and metastatic processes of tumors, as well as for the resistance to chemotherapeutic agents [80,81]. Among researchers’ goals, identifying the signature and eradication of CSCs by using targeted therapy is the biggest challenge. Pan and co-workers investigated the role of PB on human tongue squamous carcinoma cells [82]: PB promoted cell apoptosis by increasing ROS levels and, on the other hand, it downregulated the expression of stemness markers, such as octamer-binding transcription factor 4 (Oct4), sex-determining region Y-box 2 (Sox2), Nanog and Polycomb complex protein Bmi-1 [82]. Aldehyde dehydrogenase (ALDH) is highly expressed in cells including CD 34^+^ cells, c-kit^+^ cells, CD133^+^ cells, and lineage-antigen negative (Lin-) cells, and its expression has been well described in normal and cancer precursor cells of various lineages [83]. Interestingly, PB exerted a selective activity against ALDH1^+^ cells, which is also specifically expressed by a breast CSC subpopulation [84]. Here, PB reduced stem cell proliferation and the formation of the mammosphere. PB treatment inhibited the Wnt/beta-catenin pathway, blocking the epithelial–mesenchymal transition in breast CSCs. These data were also confirmed on prostate CSCs [85], where PB downregulated fibroblast growth factor (FGF)2 expression, as well as Nanog, ALDH1, and Oct4. These results have been confirmed in vivo on orthotopic xenograft nude mice [86]. All these data are in line with the previous findings [82,84,85].

The putative role of PB in modulating the biological properties of stem cells destined for tissue homeostasis is still unknown. Stem cell properties important for regenerative medicine applications. However, they need to be expanded in vitro to obtain a sufficient number of cells for transplantation. During the in vitro culturing, stem cells undergo replicative senescence [87,88], a process that can be triggered by elevated ROS levels [89,90]. In 2013, a protective role of PB against senescence in human amniotic stem cells was proposed [89]. PB, as an antioxidant agent, reduced senescence by inhibiting NADPH oxidase 4 (Nox4) activity and ROS production, as well as by enhancing stem cell proliferation [91]. PB at 2 µM showed a protective effect, while at higher doses it was cytotoxic [91]. The anti-senescent role of PB was also investigated on hair follicle dermal papilla (DP) cells [92]. DP cells are responsible for hair growth by secreting growth regulatory factors, such as FGF-7/keratinocyte growth factor (KGF) and insulin-like growth factor-1 (IGF-1). Moreover, DP cells seem to play a key role in the development of androgenic alopecia by secreting TGF-β, which negatively regulates hair follicle development [93]. PB treatment promoted DP cell proliferation and downregulated the expression of 5α-reductase type II (SRD5A2), an enzyme that works in senescence and hair follicle development [92]. Thus, PB could be implied in the treatment of androgenic alopecia.

All the reviewed effects of PB are summarized in the table below (Table 1).

## 5. Discussion

**Figure 1:**
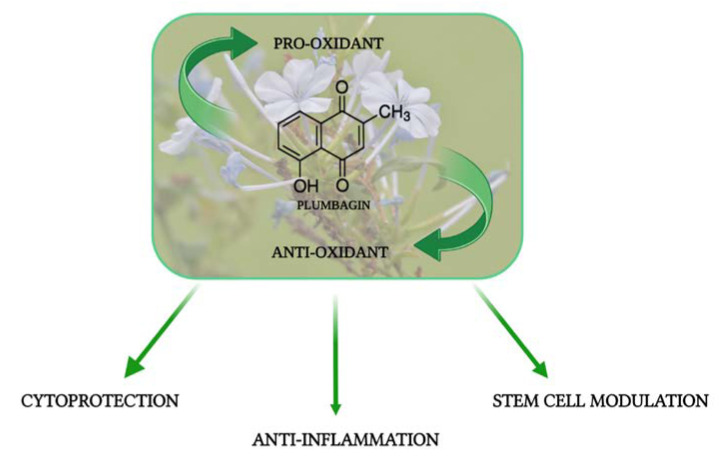
Biological activities related to Plumbagin. (Created with BioRender.com).

Natural quinones, occurring not only in plants but also in animals, have garnered attention due to their pharmacological properties and potential therapeutic significance. Drugs having quinone moiety such as anthracyclines have been used in cancer therapy revolutionizing the clinical practice. Plumbagin (PB), a biocompound occurring in many plant families which showed many interesting biological properties (Figure 1), is a member of the naphthoquinones, the most prominent type of quinones [1].

On relevance, being a plant-derived small compound, PB should not be specifically delivered, but a simple supplementation and administration can be effective to produce changes in cells. Beneficial effects can be found in how PB indirectly influences the immune system mainly by owning protective effects against many cell types. The PB capacity of promoting the downregulation of inflammation in different pathological models [18,19,20] is a result not only of its intrinsic anti-inflammatory activity but also of its antimicrobial and antifibrotic effects, in particular, in the pathologies that involve an infectious pathophysiological background.

The influence of PB on stem cell features [91] should be subjected to investigation due to the relevance of clinical research on these cells. If stem cell homeostasis is impaired, many tissues will continue to suffer chronic inflammation insults. The oxidative stress balance appears to be critical in vivo to maintain stem cell properties and retain them in vitro. Small molecules and antioxidants are usually added to stem cell media to improve ex-vivo culture conditions, for maintaining proliferation and prolonging the stemness. On the contrary, CSCs can be a target for many pro-oxidant molecules to avoid metastasis development and drug resistance. Fortunately, the scientific community is presenting encouraging evidence in searching for both these respective effects of PB on normal and cancer stem cells.

Since, the antimicrobial effects have been deeply demonstrated [1,67], not only the biomedical field can take advantage of PB, but it can also be a promising tool if applied in other health relative fields, such as in food packaging safety.

Lastly, considering the antimicrobial effect and wondering about the stem cell modulation, PB may be soon become a valid molecule to be investigated in the regenerative medicine field. In this context, PB may be employed for biomaterial functionalization, thus evolving into a useful tool for drug delivery in future biomedical clinical applications.

Of course, there are some limitations in the use and supplementation of phytochemicals like PB, even though researchers usually and successfully showed a broad spectrum of applications and advantages for inflammatory-based pathologies. As with many phytochemicals, PB has a double face, as pro-oxidant and anti-oxidant, thus its effects on cells and tissues have to be carefully considered, in terms of dosage and in vitro conditions (pH, media composition, oxidative stress, and oxygen levels), because they can determine an unexpected biological effect by revealing concealed mechanisms of action. In this context, it will also be important, before any translational application in the clinical field, to find bioavailable as well as safe doses without complications for the patients. In the future, further chemical modifications of the PB may allow some wanted specificities for targeting single cell types or increase its bioavailability for in vivo studies.

## Figures and Tables

**Table 1:** Summary of the biological effects of PB.

Cytoprotective Activity			
Biological Effect	In Vitro	In Vivo	Ref.
↑ Cell viability ↑ SOD, CAT, GST, GPx activity ↑ Nrf-2 expression ↓ Cas3, 8, 9	✓		[21,22,23,24]
↓ Bax ↑ Bcl2	✓	✓	[26,27]
↑ Mitochondrial membrane potential ↓ Apoptosis	✓		[24,28,29]
↑ Liver regeneration ↑ Autophagy	✓	✓	[30]
↓ LDH, AST, CK ↑ pTEN ↓ PI3K/Akt		✓	[34]
↓ ROS levels ↑ LC3A II/LC3 I ratio	✓		[35]
Anti-inflammatory activity			
↑ ROS levels Inactivating Nf-kB	✓		[19,20,37]
↑ Cell viability ↓ IL6, IL1 β, NO		✓	[18,20,39]
Blocking PKM2 ↓ IL1 β, NO, HMGB1	✓		[38]
↑ iNOS, IL1 α, G-CSF, IL12, MCP1, MCP5 Inactivating NLRP3-induced inflammasome	✓		[28,40,42]
↑ GSH, GPx	✓		[26]
Inactivating IRAK1	✓		[43]
↓ ROS levels, lipid peroxidation ↓ iNOS, IL6, IL18, TNF α ↑ Nrf2	✓		[21,45]
↓ RANKL ↑ OPG	✓		[52]
Inhibiting NIF ↑ Runx2, OCN, OPN, ALP → Decreasing osteoporosis	✓	✓	[24,57]
Antifibrotic activity			
↓ ALT, AST, IL6, TNF α ↑ SOD, GSH, GPx activity ↓ TGF1- β, Collagen III, α-SMA	✓	✓	[30,31,59,61,62]
Inactivating EGFR/STAT3 signaling		✓	[60]
Inhibiting TGF1- β/mTOR/Akt pathways	✓	✓	[64]
Antibacterial and antiparasitic activity			
↑ Intracellular uptake of gentamicin	✓		[70]
Inhibiting DNA gyrase activity	✓		[73]
↑ Membrane permeability and drug effect	✓		[71,74]
Inhibiting ThyX activity	✓		[75]
↓ Mitochondrial number Altering mitochondrial membrane potential	✓		[76,77]
↓ Plasmodial infection and propagation	✓	✓	[78]
Stem cells and cell senescence			
↑ ROS levels ^1^ ↓ Oct4, Sox2, Nanog, BMI-1 ^1^	✓		[82]
↓ Proliferation ^1^ ↓ FGF2, Oct4, Nanog, ALDH1 ^1^	✓	✓	[84,85,86]
↓ ROS levels ^2^ ↑ Proliferation ^2^ Inhibiting NOX4 activity	✓		[91]
↑ Proliferation ^2^ ↓ SRD5A2 → Decreasing senescence ^2^	✓		[92]

^1^ Cancer stem cells. ^2^ Normal stem cells. SOD, Superoxide dismutase; CAT, Catalase; GST, Glutathione S-transferase; GPx, Glutathione peroxidase; Nrf-2, Nuclear factor erythroid 2-related factor; Cas 3, 8, 9, Caspase 3, 8, 9; Bax, Bcl-2 associated X-protein; Bcl2, B-cell lymphoma 2; LDH, Lactate dehydrogenase; AST, Aspartate aminotransferase; CK, Creatine kinase; pTEN, Phosphatase and tensin homolog; PI3K/Akt, Phosphoinositide-3-kinase/protein kinase B (Akt); ROS, reactive oxygen species; LC3A II, Microtubule-associated protein light chain 3 II; Nf-kB, Nuclear factor-kB; IL6, Interleukin 6; IL1 β, Interleukin 1 β; NO, Nitric oxide; PKM2, Pyruvate kinases M2; HMGB1, High mobility group box 1; iNOS, Inducible nitric oxide synthase; IL1 α, Interleukin 1 α; G-CSF, Granulocyte-colony stimulating factors; IL12, Interleukin 12; MCP1, Monocyte chemoattractant protein 1; MCP5, Monocyte chemoattractant protein 5; NLRP3, NRL family pyrin domain containing 3; GSH, Glutathione; IRAK1, Interleukin-1-receptor-associated kinase; IL18, Interleukin 18; TNF α, Tumor necrosis factor α; RANKL, RANK-ligand; OPG, Osteoprotegerin; NIF, Neutrophil inhibitory factor; Runx2, Runt-related transcription factor 2; OCN, Osteocalcin; OPN, Osteopontin; ALP, Alkaline phosphatase; ALT, Alanine transaminase; TGF1-β, Transforming growth factor 1-β; α-SMA, α-smooth muscle actin; EGFR/STAT3, Epidermal growth factor receptor/Signal transducer and activator of transcription 3; mTOR, Mammalian target of rapamycin; ThyX, Thymidylate synthase; Oct4, Octamer-binding transcription factor 4; Sox2, Sex determining region Y-box 2; Nanog, Homeobox protein Nanog; ALDH1, Aldehyde dehydrogenase 1; NOX4, NADPH oxidase 4; SRD5A2, 5α-reductase type II. ↑ means “increase in”, ↓ means “decrease in”, → means “imply”.

## Data Availability

Not applicable.
